# Transactivation of the EGF receptor as a novel desensitization mechanism for G protein-coupled receptors, illustrated by dopamine D2-like and β_2_ adrenergic receptors

**DOI:** 10.1186/s11658-024-00652-z

**Published:** 2024-10-28

**Authors:** Dooti Kundu, Xiao Min, Shujie Wang, Lulu Peng, Xinru Tian, Mengling Wang, Kyeong-Man Kim

**Affiliations:** Department of Pharmacology, College of Pharmacy, Chonnam National University, Gwang-Ju, 61186 Korea

**Keywords:** GPCR, EGFR, Transactivation, Desensitization, Arrestin, Deubiquitination

## Abstract

Transactivation of epidermal growth factor receptors (EGFR) provides intricate control over multiple regulatory cellular processes that merge the diversity of G protein-coupled receptors (GPCRs) with the robust signaling capacities of receptor tyrosine kinases. Contrary to the typical assertions, our findings demonstrate that EGFR transactivation contributes to the desensitization of GPCRs. Repeated agonist stimulation of certain GPCRs enhanced EGFR transactivation, triggering a series of cellular events associated with GPCR desensitization. This effect was observed in receptors undergoing desensitization (D_3_R, K149C-D_2_R, β_2_AR) but not in those resistant to desensitization (D_2_R, C147K-D_3_R, D_4_R, β_2_AR mutants lacking GRK2 or GRK6 phosphorylation sites). The EGFR inhibitor AG1478 prevented both desensitization and the associated cellular events. Similarly, these cellular events were also observed when cells were treated with EGF, but only in GPCRs that undergo desensitization. These findings suggest that EGFR transactivation diversifies pathways involved in ERK activation through the EGFR signaling system and also mediates GPCR desensitization. Alongside the widely accepted steric hindrance model, these findings offer new insights into understanding the mechanisms of GPCR desensitization, which occurs through complex cellular processes.

**Supplementary Information:**

The online version contains supplementary material available at 10.1186/s11658-024-00652-z.

## Introduction

Desensitization refers to the gradual reduction in receptor responsiveness resulting from repeated or prolonged exposure to agonists [1, 2]. It is a fundamental regulatory mechanism in biology that ensures appropriate cellular responses to signaling molecules, prevents overstimulation, and maintains cellular homeostasis [3, 4].

In the classical model of homologous desensitization of G protein-coupled receptors (GPCRs), GPCRs activated by agonists not only stimulate G proteins but also are recognized and phosphorylated by GPCR kinases (GRKs) [5, 6]. Subsequently, arrestins bind to the phosphorylated GPCRs [7] and this binding prevents the GPCRs from coupling with G proteins (i.e., leading to desensitization) owing to the overlapping interfaces between the G protein-binding sites on GPCRs and the arrestin-binding sites [8, 9].

Recently, we introduced a novel desensitization model on the basis of comparative studies involving several GPCRs undergoing desensitization and those that do not. According to this model, the desensitization of GPCRs is facilitated by the deubiquitination of arrestins through a signaling cascade that includes Src, PDK1 (3-phosphoinositide-dependent protein kinase 1), Akt, and USP33 (ubiquitin-specific peptidase 33). Src phosphorylates PDK1 at S241 to activate it, the activated PDK1 phosphorylates T308 of Akt, and the phosphorylated Akt subsequently phosphorylates USP33. The activated USP33 is recruited near the plasma membrane and associates with the arrestins to deubiquitinate them. Deubiquitinated arrestins form a complex with Gβγ, which subsequently undergoes importin-mediated entry into the nucleus. This process eventually sequesters Gβγ from the GPCRs, resulting in signaling attenuation [10, 11]. However, the specific pathway through which GPCRs are linked to the signaling cascade that results in desensitization remains unclear.

GPCRs and receptor tyrosine kinases (RTKs) are the major classes of cell surface receptors that employ their own established signaling pathways. It has been reported that agonists of some GPCRs can activate RTKs in the absence of growth factors [12]. These observations have led to the existence of the “transactivation” or “cross-communication” concept, which designates a phenomenon in which a given receptor is activated by a ligand of a different class of receptor. For instance, GPCRs are recognized for their involvement in the RTK signaling pathway, as seen in the activation of ERK [13].

The EGFR, a member of the ErbB family of RTKs, is endogenously expressed in various cell types, including neurons found in the cerebral cortex, cerebellum, hippocampus, and other areas of the brain [14, 15]. EGFR is connected to multiple signal transduction cascades, including the MAPK, Akt, and JNK pathways, which ultimately promote DNA synthesis and cell proliferation [16]. Thus, transactivation is anticipated to provide a mechanism for expanding both the quantity and scope of signaling networks within cells. This occurs through the integration of diverse combinations of GPCRs and their respective ligands with the extensive signaling networks facilitated by RTKs.

EGFR transactivation induced by GPCR can occur through both ligand-dependent and ligand-independent mechanisms [17]. The ligand-dependent pathway involves the cleavage of heparin-binding (HB)-EGF mediated by matrix metalloprotease (MMP) or a disintegrin and metalloprotease (ADMA) [18, 19]. On the other hand, the ligand-independent pathway entails the activation of various intracellular signaling pathways that lead to tyrosine phosphorylation in the cytosolic domain of EGFR [20].

While investigating the signal transduction pathways responsible for ERK activation via GPCRs, we noted that EGFR participates in ERK activation for certain GPCRs, but not others. Upon examining the traits of these GPCRs across both groups, we observed consistent patterns in EGFR involvement and their desensitization characteristics. Furthermore, we identified that the signaling components crucial to the novel desensitization model we recently proposed are all situated downstream of EGFR [16, 21, 22].

To explore the relationship between GPCR desensitization and EGFR transactivation, this study concentrated on dopamine D2-like receptors (D_2_R, D_3_R, D_4_R) and the β_2_ adrenoceptor. These receptors have been thoroughly examined for their desensitization traits [1, 23–26], including various mutants with altered desensitization characteristics [27]. Only GPCRs with desensitization properties transactivated EGFR, and the transactivation intensity was significantly enhanced under desensitization conditions. Similarly, direct stimulation of EGFR exhibited a comparable pattern, reproducing the signaling cascade involved in GPCR desensitization. These findings, along with the widely accepted steric hindrance hypothesis, propose new features of GPCR desensitization that can manifest through various aspects of cellular contexts.

## Materials and methods

### Reagents

Dopamine, (−) quinpirole, isoproterenol, forskolin, EGF, AG1478, agarose beads coated with monoclonal antibodies against FLAG epitope, rabbit anti-FLAG M2 antibodies (AB_439687), rabbit antibodies against green fluorescent protein (GFP; AB_439690), and HA antibodies (AB_2610070) were purchased from Sigma-Aldrich Chemical Co. (St. Louis, MO, USA). Goat anti-mouse Alexa Fluor® 555 (AB_2535844), anti-rabbit Alexa Fluor® 555 (AB_2535849), anti-rabbit horseradish peroxidase (HRP)-conjugated secondary antibodies (AB_10960844), and DAPI were purchased from Thermo Fisher Scientific (Waltham, MA, USA). Antibodies to β-actin (AB_10950489), phospho-T308-Akt (AB_331170), phospho-S241-PDK1 (AB_2161134), phospho-Y416-Src (AB_331697), and arrestin2/3 (AB_10547883) were purchased from Cell Signaling Technology (Danvers, Massachusetts, USA). Antibodies to phospho-ERK1/2 (AB_1125801) and ERK2 (AB_2141292) were obtained from Santa Cruz Biotechnology (Santa Cruz, CA, USA). Anti-mouse HRP-conjugated secondary antibodies (AB_10015289) were obtained from Jackson ImmunoResearch (West Grove, PA, USA).

### Cell culture and transfection

Human embryonic kidney 293 (HEK-293, CVCL_0045) cells were obtained from the American Type Culture Collection (Manassas, VA, USA) and cultured in minimal essential medium supplemented with 10% fetal bovine serum (FBS; Thermo Fisher Scientific), 100 U/mL penicillin, and 100 μg/mL streptomycin in a humidified atmosphere containing 5% CO_2_. GRK2-KD and GRK6-KD cells were established by stable transfection of shRNA in pLKO.1 [28]. The control knockdown (Con-KD) cells for GRK2 or GRK6 were established by stable transfection of a plasmid containing scrambled shRNA in pLKO.1.

### Plasmid constructs

Dopamine D_2_R, D_3_R, D_4_R, K149C-D_2_R, C147K-D_3_R, and β_2_AR have been described in previous studies [25, 29]. β_2_AR mutants with alterations at the consensus phosphorylation sites targeted by GRK2 and GRK6 [26], termed GRK2-KO-β_2_AR and GRK6-KO-β_2_AR respectively, were generated through site-directed mutagenesis. HA-Src, FLAG-PDK1, HA-USP33, FLAG-arrestin3, EGFR-GFP, GRK2-CT, GFP-Gβ1, Gγ2, and FLAG-Akt were described previously [11, 30].

### Luciferase report gene assay

Cellular cyclic adenosine monophosphate (cAMP) determinations were based on an indirect method using a reporter plasmid containing the firefly luciferase gene under the control of multiple cAMP-responsive elements (CREs) and pRL-TK as a control vector (Promega Corp., Madison, WI), which has previously been used for determining D_1_R, D_2_R, and D_3_R signaling [31, 32]. This method produces essentially the same results as those obtained on the basis of the use of a direct assay in which [^3^H]-cAMP accumulation is determined via sequential chromatography [31, 33]. Data were normalized by expressing cAMP levels as a percentage of the forskolin-stimulated cAMP for each experiment for the Gi/o-coupled receptors. For the β_2_ adrenoceptors that couple to Gs, the rise in agonist-induced reporter gene expression was determined in the absence of forskolin treatment, with each dose’s reaction expressed as a percentage of the maximum response. Dose–response curves were constructed using GraphPad Prism (Graph Pad Software; San Diego, CA, USA).

### Induction of desensitization

Desensitization was induced as previously described [25]. Briefly, cells expressing the corresponding D2-like receptors or β_2_AR were treated with the agonist (quinpirole or isoproterenol) for 1–5 min (initial treatment) to trigger desensitization and then washed three times with serum-free medium (5 min per wash) at 37 °C to remove the receptor-bound agonist. The cells were then rechallenged with the agonist to induce a secondary response. The development of desensitization was determined by comparing the amplitudes of the secondary response in vehicle- or agonist-pretreated cells. Quin(+) and Quin(w/ +) represent the cells treated with quinpirole and washed/rechallenged, respectively.

### Immunoprecipitation and immunoblotting

The cells were first lysed by placing them on a rotating wheel in ice-cold radioimmunoprecipitation assay (RIPA) buffer [containing 50 mM of Tris, 150 mM of NaCl, pH 8.0, 0.5% deoxycholate, 1% NP-40, 0.1% sodium dodecyl sulfate (SDS)] for 1 h at 4 °C. After lysing, the cell extracts were clarified by centrifugation (at 14,000 *g*) in a microcentrifuge at 4 °C, and the resulting supernatant was combined with 25 μL of 50% agarose beads coated with anti-M2 FLAG antibody. These immunoprecipitated samples were then incubated overnight at 4 °C with rotation. Following this, the agarose beads were washed three times (each for 5 min) with ice-cold washing buffer (composed of 50 mM Tris, pH 7.4, 137 mM NaCl, 10% glycerol, 1% NP-40). Subsequently, the immunoprecipitates and the original cell lysates were subjected to analysis using SDS-polyacrylamide gel electrophoresis (PAGE) and immunoblotting techniques. The resulting blots were quantified using the ChemiDoc MP imaging system (BioRad, Hercules, California, USA).

### Detection of arrestin3 ubiquitination

FLAG-arrestin3 and HA-Ub were used to co-transfect HEK-293 cells expressing D_3_R. Cells were treated with 10 ng/mL EGF for 20 min or 5 min and washed/rechallenged (w/ +). The cell lysates were solubilized in RIPA buffer containing 1 mM of sodium orthovanadate, 1 mM of sodium fluoride, 10 mM of n-ethylmaleimide, 5 μg/mL of leupeptin, 5 μg/mL of aprotinin, and 2 mM of phenylmethylsulphonyl fluoride, after which they were immunoprecipitated with FLAG beads. The eluents were analyzed using immunoblotting.

### Immunocytochemistry

Cells were fixed with 4% paraformaldehyde in phosphate-buffered saline (PBS) for 10 min at 20 °C, washed three times with PBS, and then incubated with blocking buffer [1% FBS and 1% bovine serum albumin (BSA) in PBS] for 1 h at 20 °C. The cells were then incubated with antibodies against arrestin3 or HA (1:1000 dilution) for 1 h at 20 °C, followed by washing with PBS and incubation with the secondary antibodies (Alexa 555-conjugated antibodies at 1:500 dilution). The immune-stained cells were subsequently mounted on slides using Vectashield (Vector Laboratories; Burlingame, CA, USA) and imaged using a laser scanning confocal microscope (TCS SP5/AOBS/Tandem; Leica, Jena, Germany). The images were analyzed using the Fiji version of the image processing software ImageJ and colocalization was analyzed on the basis of Pearson’s correlation coefficient (γ value).

### Statistical analysis

The statistical analysis was carried out using independent values corresponding to data obtained from different immunoblots or assays. For immunoblots, to mitigate variability across experiments, the control group was normalized to 1, with other values presented as fold changes. The analysis was interpreted using GraphPad Prism 5 software (GraphPad Software Inc., San Diego, CA, USA). All data were expressed as the mean ± standard deviation (SD). Statistical significance was analyzed by using a paired two-tailed Student’s *t-*test for the two groups or one-way analysis of variance (ANOVA) with Tukey’s post hoc for multiple groups.

## Results

### Transactivation of EGFR links the desensitization cascade of G protein-coupled receptors

In the desensitization model we introduced recently, a sequence of signaling pathways orchestrates receptor desensitization. In essence, arrestins associated with the receptor undergo deubiquitination under desensitization conditions (repeated agonistic stimulation) [10] via the Src, PDK, Akt, and USP33 signaling pathways. Subsequently, deubiquitinated arrestins form a complex with Gβγ and migrate into the nucleus [11]. Through preliminary experiments and a literature review, we recognized that EGFR is involved in mediating this signaling cascade.

As the first step to prove our hypothesis, we determined whether EGFR is involved in the desensitization of G protein-coupled receptors (GPCRs) by testing whether AG1478, an EGFR inhibitor [34], blocks the GPCR desensitization and the regulatory cascade that mediate the desensitization.

For Akt phosphorylation induced by EGFR stimulation, AG1478 almost completely inhibited it with 200 nM treatment for 5 min or 10 nM treatment for 30 min (Fig. S1A and S1B). Regarding ERK phosphorylation induced by EGFR, AG1478 inhibited it in a time- and dose-dependent manner, almost completely inhibiting it with 200 nM treatment for 30 min (Fig. S1C and S1D). Meanwhile, AG1478 did not affect Akt phosphorylation (Fig. S1E) or ERK phosphorylation (Fig. S1F) caused by PDGF stimulation, which utilizes a similar signaling pathway to EGFR, under the same conditions. These results indicate that AG1478 has high selectivity for EGFR and effectively inhibits most cellular events triggered by EGFR when treated with 200 nM for 30 min.

**Fig. 1:**
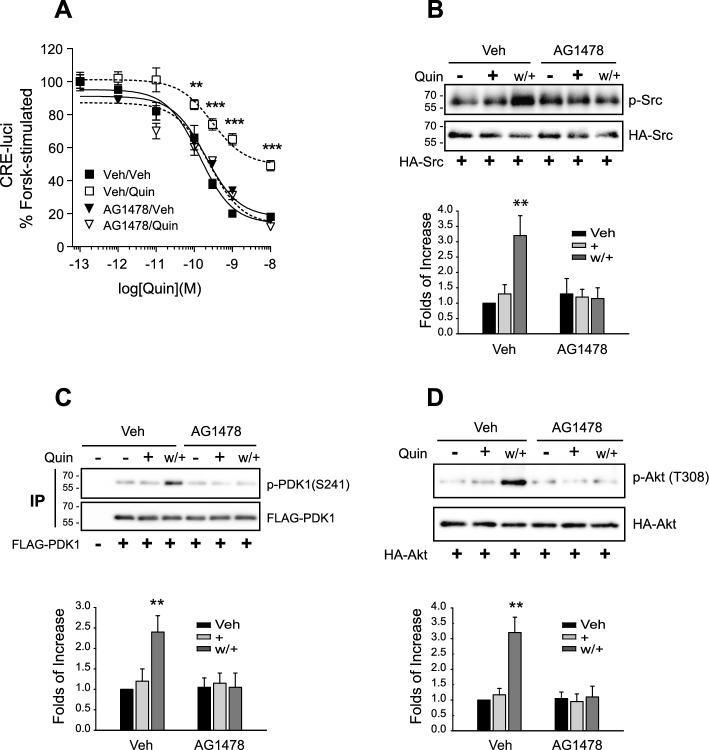
Transactivation of EGFR is involved in D_3_R desensitization. **A** HEK-293 cells expressing D_3_R (1.7–2.3 pmol/mg protein) were pretreated with vehicle or 200 nM AG1478 for 30 min, followed by desensitization induction (w/+). Quin (+) and Quin (w/+) represent the cells treated with 100 nM of quinpirole for 5 min and washed/rechallenged, respectively. **p < 0.01, ***p < 0.001 compared with other groups at 10^–10^–10^−8^ M Quin (*n* = 3). **B** Cells were transfected with HA-Src. The cell lysates were immunoblotted with antibodies against p-Src (Y416) and HA. **p < 0.01 compared with other groups except Veh/AG1478 group; ^#^*p* < 0.05 compared with Veh/AG1478 group (*n* = 4). **C** HEK-293 cells expressing D_3_R were transfected with FLAG-PDK1. The cell lysates were immunoprecipitated with FLAG beads. IPs were immunoblotted with antibodies against p-PDK1 (S241) and FLAG. **p < 0.01 compared with Veh/Veh group; ^#^*p* < 0.05 compared with other groups except Veh/Veh group (*n* = 4). **D** HEK-293 cells expressing D_3_R were transfected with HA-Akt. The cell lysates were immunoblotted with antibodies against p-Akt (T308) and HA. **p < 0.01 compared with other groups (*n* = 4)

**Fig. 2:**
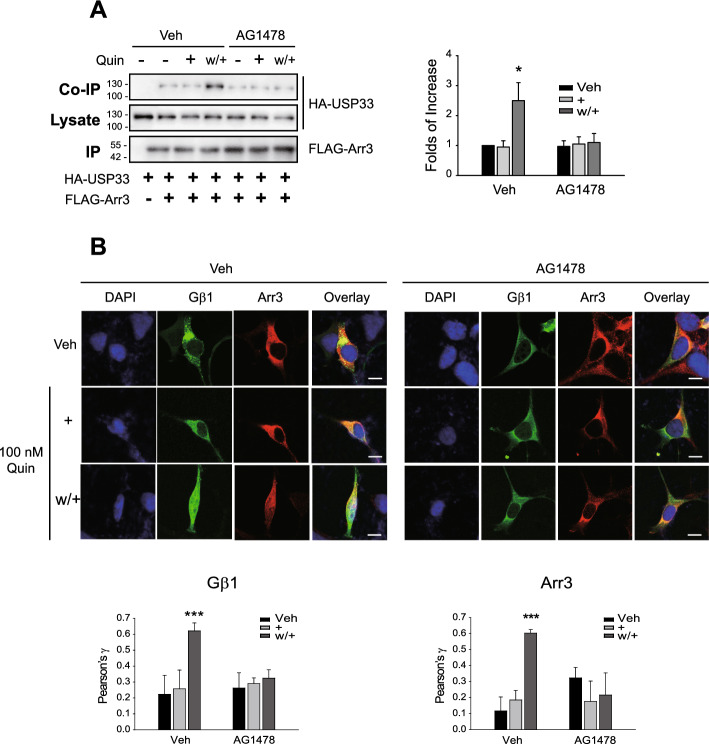
Transactivation of EGFR is involved in the sequestration of Gβγ into the nucleus. **A** HEK-293 cells expressing D_3_R (1.8–2.1 pmol/mg protein) were transfected with HA-USP33 and FLAG-arrestin3. The cell lysates were immunoprecipitated with FLAG beads. Co-IP/lysate and IPs were immunoblotted with antibodies against HA and FLAG, respectively. **p < 0.01 compared with other groups (*n* = 4). **B** HEK-293 cells expressing D_3_R (1.9 pmol/mg protein) were transfected with GFP-Gβ1, Gγ2, and arrestin3. The cells were labeled with arrestin2/3 antibodies (1:1000), followed by Alexa 555-conjugated secondary antibodies (1:500). Scale bars represent 10 μm. The Pearson correlation coefficient (γ value) represents the degree of colocalization between DAPI and GFP-Gβ1 (bar graphs on the left) or DAPI and arrestin3 (bar graphs on the right). ***p < 0.001 compared with other groups (*n* = 5)

As shown in Fig. 1A, pretreatment with AG1478 prevented the desensitization of D_3_R (Fig. 1A). AG1478 also inhibited the phosphorylation of Src at Y416 (Fig. 1B), PDK1 at S241 (Fig. 1C), and Akt at T308 (Fig. 1D). The desensitization-induced increase in the interaction between Akt and USP33 (Fig. 2A), and the nuclear translocation of Gβγ and arrestin (Fig. 2B) were also inhibited by pretreatment with AG1478.

Thus, the receptor desensitization principle established on the basis of our studies of D2-like receptors is likely to occur through transactivation of EGFR.

### Direct stimulation of EGFR activates cellular events that mediate D_3_R desensitization

**Fig. 3:**
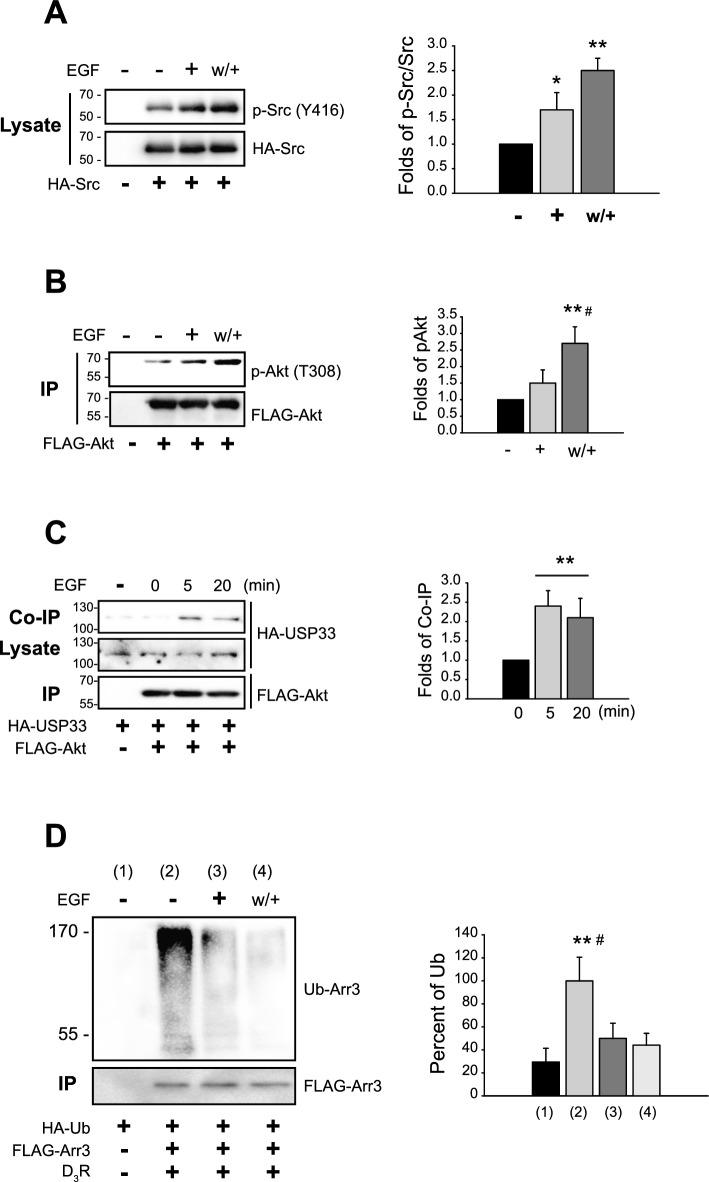
Direct stimulation of EGFR induces the cellular cascade involved in GPCR desensitization. **A**, **B**, **D** EGF (+) represents the cells treated with 10 ng/mL EGF for 20 min; EGF (w/+) represents the cells treated with EGF for 5 min, washed, and rechallenged with EGF for 5 min. A HEK-293 cells transfected with HA-Src. Cell lysates were immunoblotted with antibodies against p-Src (Y416) and HA. *p < 0.05, **p < 0.01 compared with the “−” group (*n* = 4). **B** HEK-293 cells transfected with FLAG-Akt. Cell lysates were immunoprecipitated with FLAG beads, and IPs were immunoblotted with antibodies against p-Akt (T308) and FLAG, respectively. **p < 0.01, ^#^*p* < 0.05 compared with the “−” and “ + ” group, respectively (*n* = 3). **C** HEK-293 cells transfected with HA-USP33 and FLAG-Akt were treated with 10 ng/mL EGF for 5 min or 20 min. Cell lysates were immunoprecipitated with FLAG beads. Co-IP/lysate and IPs were immunoblotted with antibodies against HA and FLAG. **p < 0.01 compared with the “0” min group (*n* = 4). **D** HEK-293 cells expressing D_3_R were transfected with HA-Ub and FLAG-arrestin3. Cells were treated with EGF, followed by the ubiquitination assay. The lower panel (IP) displays the immunoprecipitated total FLAG-arrestin3, while the upper panel shows the ubiquitin-conjugated arrestin3. **p < 0.01, ^#^*p* < 0.05 compared with “1” and “3 or 4” groups, respectively (*n* = 3)

If EGFR is indeed a mediator of GPCR desensitization, treatment with EGF should induce identical cellular events as when GPCR desensitization is induced. Therefore, we investigated whether the desensitization-mediated cellular cascade was triggered by prolonged EGF treatment (20 min) or by repeated EGF treatments (w/+), both of which are conditions known to induce receptor desensitization. Indeed, biochemical changes in cellular components constituting the GPCR desensitization cascade were evoked when cells were treated with EGF, such as phosphorylation of Src (Fig. 3A), Akt (Fig. 3B), the interaction between Akt and USP33 (Fig. 3C), which culminated in the deubiquitination of arrestins (Fig. 3D). As reported in previous studies, cells expressing GPCRs with desensitization characteristics exhibit high levels of arrestin3 ubiquitination in the basal state (Fig. 3D, 2nd lane) [29, 35].

Under desensitization conditions, however, this ubiquitination decreases (3rd and 4th lane) [10, 11]. During the ubiquitination of a protein, a single ubiquitin molecule (approximately 8.6 kDa) is initially attached to a specific ubiquitination site on the target protein [36]. This process is typically repeated, with additional ubiquitin molecules either linking sequentially to the previously attached ubiquitin or attaching to another ubiquitination site on the target protein. As a result, the molecular weight of the target protein increases in a heterogeneous manner, depending on the number of ubiquitin molecules added [37]. Consequently, ubiquitinated proteins often appear as diffuse bands in immunoblot analysis. Therefore, arrestin, which has a predicted molecular weight of about 47 kDa, appears diffusely in the 50–170 kDa range on SDS-PAGE.

### GPCRs that undergo desensitization activate ERK via the transactivation of EGFR

In light of the findings depicted in Figs. 1, 2, and 3, which imply a relationship between GPCR desensitization and EGFR transactivation, we set out to explore whether this correlation was a common occurrence by expanding the number of cases studied.

**Fig. 4:**
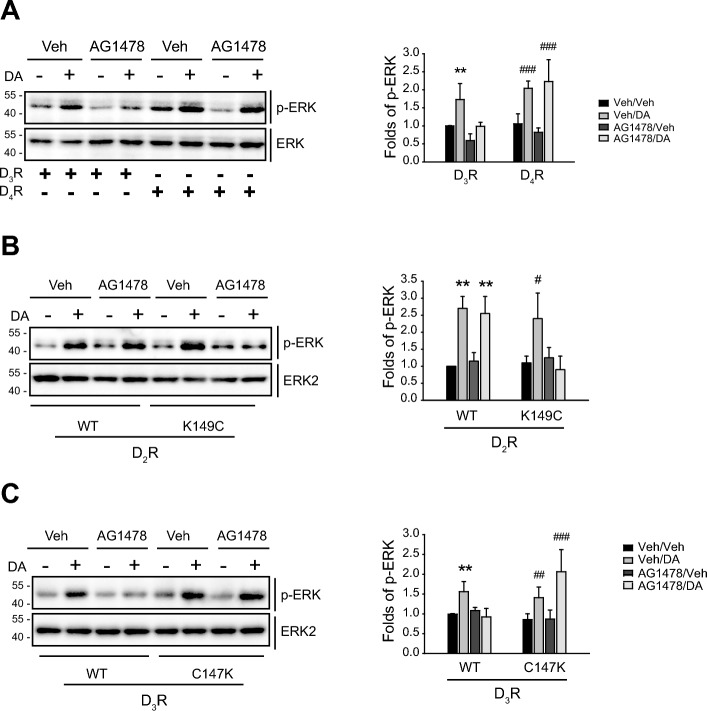
Correlation between the GPCR desensitization and transactivation of EGFR. Cells expressing D2-like receptors (1.7–2.1 pmol/mg protein) were pretreated with vehicle or 200 nM AG1478 for 30 min, followed by treatment with 1 or 10 μM DA for 5 min. The cell lysates were immunoblotted with antibodies against phospho-ERK1/2 and ERK2. **A** HEK-293 cells expressing D_3_R or D_4_R were treated with 1 μM DA. **p < 0.01 compared with other groups in D_3_R-expressing cells; ^###^*p* < 0.001 compared to their respective vehicle-treated groups in D_4_R-expressing cells (*n* = 3). **B** HEK-293 cells expressing D_2_R or K149C-D_2_R were treated with 10 μM DA. **p < 0.01 compared with the corresponding vehicle-treated groups in WT-D_2_R-expressing cells (*n* = 3); ^#^*p* < 0.05 compared with other groups of the cells expressing K149C-D_2_R (*n* = 3). **C** HEK-293 cells expressing D_3_R or C147K-D_3_R were treated with 1 μM DA. **p < 0.01 compared with other groups in WT-D_3_R-expressing cells; ^##^*p* < 0.01, ^###^*p* < 0.001 compared with the corresponding vehicle-treated groups of the cells expressing C147K-D_3_R (*n* = 3)

Among three D2-like receptors (D_2_R, D_3_R, D_4_R), only D_3_R undergoes desensitization [25, 27, 29]. Pretreatment with AG1478 blocked the ERK activation mediated by D_3_R but not by D_4_R (Fig. 4A) or D_2_R (Fig. 4B). These results suggest that only D2-like receptors that undergo desensitization might transactivate EGFR. Additionally, D_2_R and D_3_R mutants with different desensitization properties were utilized to substantiate this assertion. Previous studies have shown that a point mutation of D_2_R or D_3_R in the second intracellular loops [25, 27] alters desensitization properties. For instance, the replacement of the 149th lysine of D_2_R with cysteine (K149C) endows D_2_R with the ability to undergo desensitization. In contrast, the replacement of the 147th cysteine of D_3_R with lysine (C147K) renders D_3_R resistant to desensitization. As shown in Fig. 4B, agonist-induced ERK activation mediated by K149C-D_2_R but not WT-D_2_R-mediated ERK activation was blocked by pretreatment with AG1478; the ERK activation mediated by WT-D_3_R but not by K147C-D_3_R was inhibited by pretreatment with AG1478 (Fig. 4C).

### Direct stimulation of EGFR selectively inhibits the signaling of D2-like receptors which undergo desensitization

**Fig. 5:**
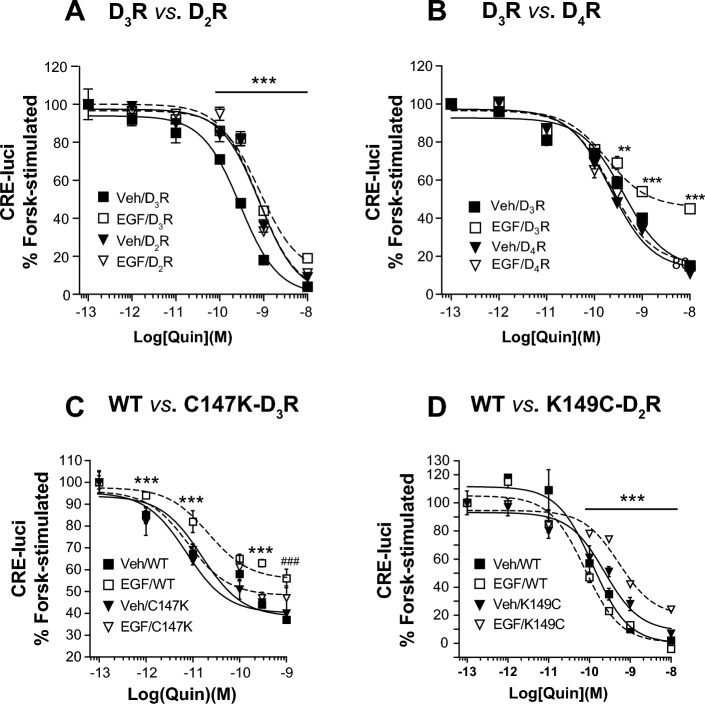
Involvement of transactivation of EGFR in the D2-like receptor desensitization. Cells expressing D2-like receptors and related mutants (1.7–2.1 pmol/mg protein) were pretreated with vehicle or 10 ng/mL EGF for 20 min, and the quinpirole-induced inhibition of the cAMP production was determined. **A** HEK-293 cells expressing D_3_R or D_2_R were used. The Veh/D_3_R group differed significantly from other groups of the cells expressing D_3_R or D_2_R at 10^–10^ to 10^−8^ M quinpirole (*p* < 0.001, *n* = 3). **B** HEK-293 cells expressing D_3_R or D_4_R were used. The EGF/D_3_R group showed a significant difference from other groups of the cells expressing either D_3_R or D_4_R at 10^−10^ M quinpirole (*p* < 0.01, *n* = 3) and 10^−9^ to 10^−8^ M quinpirole (*p* < 0.001, *n* = 3). **C** HEK-293 cells expressing WT-D_3_R or C147K-D_3_R were used. ***p < 0.001 compared with other groups in WT-D_3_R- and C147K-D_3_R-expressing cells; ^###^*p* < 0.001 compared with Veh-treated groups of the cells expressing WT-D_3_R or C147K-D_3_R (*n* = 3). **D** HEK-293 cells expressing WT-D_2_R or K149C-D_3_R were used. The EGF/K149C group differed significantly from other groups of WT-D_2_R- or K149C-D_2_R-expressing cells at 10^−10^–10^−8^ M quinpirole (*p* < 0.001, *n* = 3)

If the transactivation of EGFR by specific GPCRs is the mechanism responsible for GPCR desensitization, then direct stimulation of EGFR is expected to inhibit GPCR signaling. When EGFR was directly stimulated with EGF, signaling of D_2_R, D_4_R, and C147K-D_3_R, which do not undergo desensitization [25, 27], was not affected (Fig. 5A–C). In contrast, the signaling of D_3_R and K149C-D_2_R, which undergoes desensitization [25, 27], was inhibited (Fig. 5D).

### Correlation between the desensitization of D2-like receptors and the formation of a complex with EGFR

**Fig. 6:**
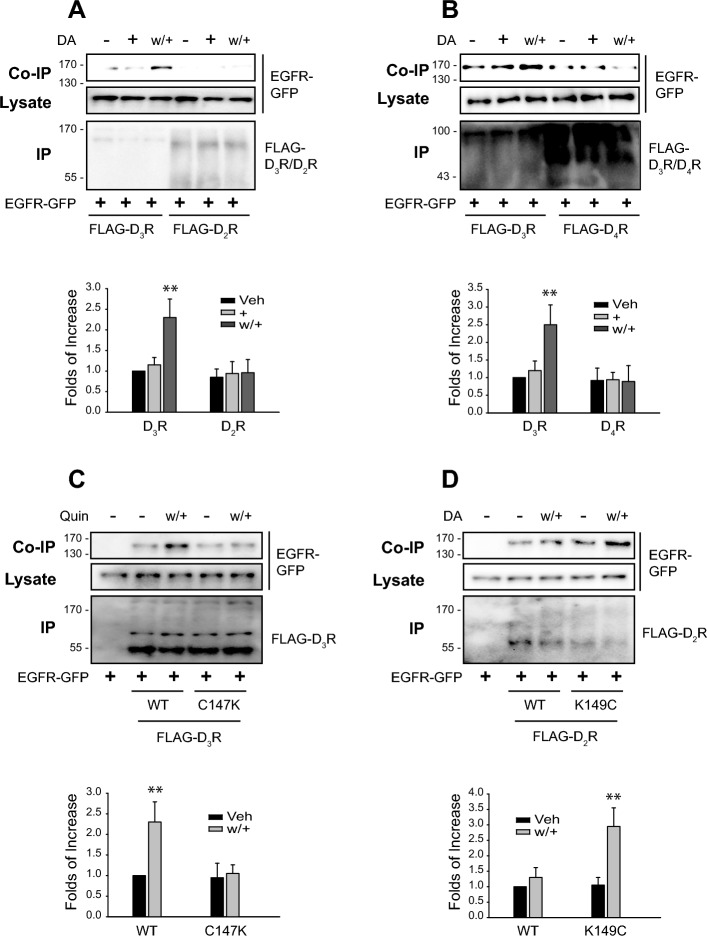
Induction of the GPCR desensitization causes the interaction between D2-like receptors. HEK-293 cells were transfected with EGFR-GFP along with FLAG-tagged D2-like receptors. “ + ” and “w/+” represent the cells treated with the corresponding agonist for 5 min and washed/rechallenged, respectively. The cell lysates were immunoprecipitated with FLAG beads. Co-IP/lysate and IP were immunoblotted with antibodies against GFP and FLAG. **A** FLAG-tagged D_2_R and D_3_R were used. Cells were treated with 10 μM of dopamine. **p < 0.01 compared with other groups of the cells expressing D_3_R or D_4_R (*n* = 3). **B** FLAG-tagged D_3_R and D_4_R were used. Cells were treated with 10 μM of dopamine. **p < 0.01 compared with other groups of the cells expressing D_3_R or D_4_R (*n* = 4). **C** FLAG-tagged WT-D_3_R and C147K-D_3_R were used. Cells were treated with 100 nM of quinpirole. **p < 0.01 compared with other groups of the cells expressing WT-D_3_R or C147K-D_3_R (*n* = 4). **D** FLAG-tagged WT-D_2_R and K149C-D_2_R were used. Cells were treated with 10 μM of dopamine. **p < 0.01 compared with other groups of the cells expressing WT-D_2_R or K149C-D_2_R (*n* = 3)

Formation of the complex between the GPCR and EGFR is one of the criteria that supports the transactivation between them [38, 39]. Thus, the transactivation of EGFR through a GPCR was further confirmed with the interaction of EGFR with the GPCR. As shown in Fig. 6A–D, the receptors that undergo desensitization (D_3_R and K149C-D_2_R), not the ones that are desensitization-resistant (D_2_R, D_4_R, and C147K-D_3_R) interacted with EGFR when they were repeatedly stimulated with 10 μM of dopamine or 100 nM of quinpirole, suggesting that only the GPCR that undergoes desensitization are closely associated with EGFR.

GPCRs appear as diffuse bands in protein gels primarily because of their complex posttranslational modifications, such as glycosylation [40] and phosphorylation, which add varying amounts of mass and can alter their migration patterns. Additionally, GPCRs often exist in multiple oligomeric states (e.g., dimers or higher-order complexes) and exhibit conformational variability, all of which contribute to a spread of molecular weights and sizes that lead to the appearance of diffuse bands rather than distinct, sharp bands [41]. Therefore, the majority of the D2-like receptors, with predicted molecular weights ranging from 45 to 50 kDa, appear diffusely in the 40–80 kDa range on SDS-PAGE [42].

**Fig. 7:**
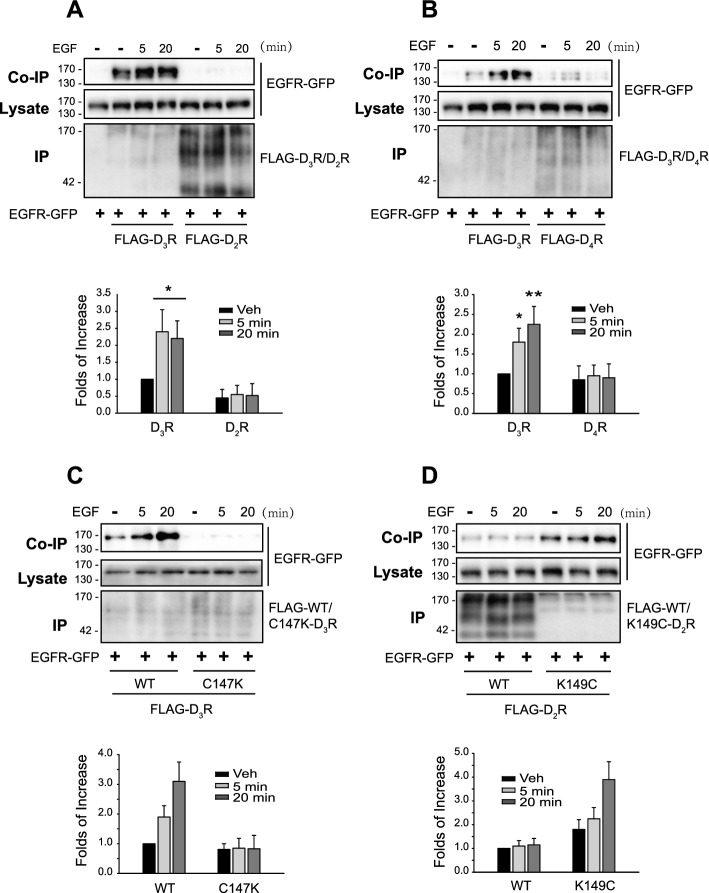
Correlation between D2-like receptor desensitization and EGF-induced GPCR-EGFR interaction. HEK-293 cells were transfected with EGFR-GFP along with corresponding FLAG-tagged D2-like receptors. Cells were treated with either vehicle or 10 ng/mL of EGF for 5 or 20 min. The cell lysates were immunoprecipitated with FLAG beads. Co-IP/lysate and IP were immunoblotted with antibodies against GFP and FLAG. **A** FLAG-tagged D_2_R and D_3_R were used. *p < 0.05 compared with other groups of the cells expressing D_2_R or D_3_R (*n* = 3). **B** FLAG-tagged D_3_R and D_4_R were used. *p < 0.05, **p < 0.01 compared with other groups of the cells expressing D_3_R or D_4_R (*n* = 3). **C** FLAG-tagged WT-D_3_R and C147K-D_3_R were used. *p < 0.05, **p < 0.01 compared with other groups of the cells expressing WT-D_3_R or C147K-D_3_R (*n* = 4). **D** FLAG-tagged WT-D_2_R and K149C-D_2_R were used. ***p < 0.001 compared with the groups of WT-D_2_R-expressing cells; ^##^*p* < 0.01 compared with other groups of K149C-D_2_R-expressing cells (*n* = 4)

The results in Figs. 4 and 5 suggest that EGFR transactivation is involved in the desensitization of D2-like receptors. Accordingly, on the basis of the findings in Fig. 6, it was anticipated that similar results would be obtained when cells were treated with EGF instead of dopamine. As expected, D_2_R, D_4_R, and C147K-D_3_R, which do not undergo desensitization, did not form a complex with EGFR when EGFR was directly stimulated with EGF (Fig. 7A–C). On the other hand, D_3_R or K149C-D_2_R, which undergoes desensitization, formed a complex with EGFR (Fig. 7C and D).

### Desensitization of β_2_ adrenergic receptors can be explained in terms of the transactivation of EGFR

**Fig. 8:**
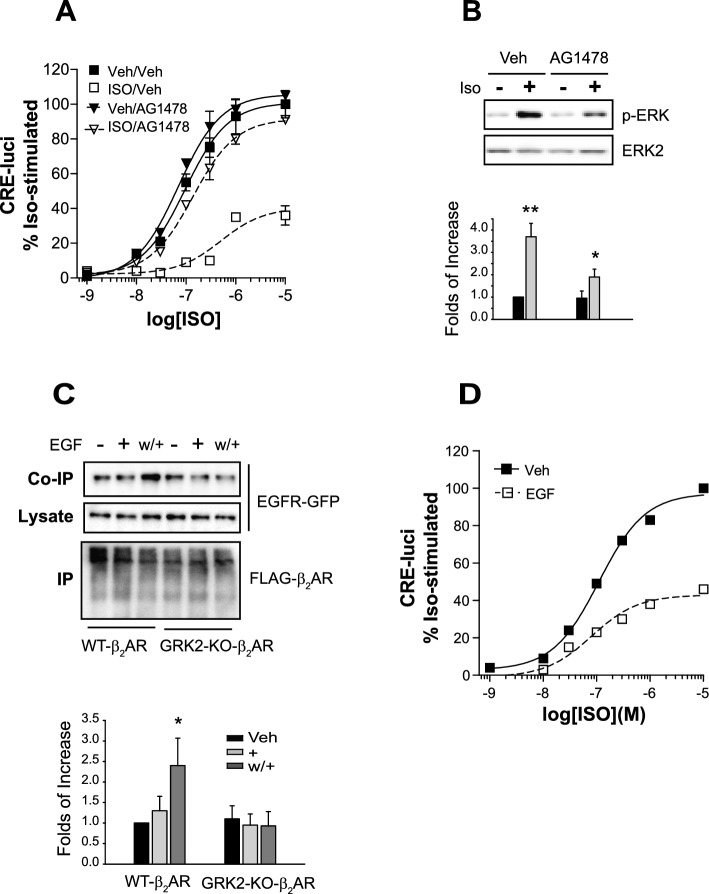
Involvement of EGFR transactivation in the desensitization of β_2_ adrenoceptors. A HEK-293 cells expressing β_2_AR were pretreated with either vehicle or 200 nM of AG1478 for 30 min, followed by treatment with 10 μM of isoproterenol for 5 min and washed/rechallenged to induce desensitization. ISO/Veh group was significantly different from other groups at 10^−8.5^–10^−5^ M isoproterenol (*n* = 3). B HEK-293 cells expressing β_2_AR were pretreated with either vehicle or 200 nM of AG1478 for 30 min, followed by treatment with 10 μM of isoproterenol for 2 min to induce the ERK activation. *p < 0.05, **p < 0.01 compared with corresponding vehicle-treated groups (*n* = 3). C HEK-293 cells were transfected with EGFR-GFP together with either FLAG-tagged WT-β_2_AR or GRK2-KO-β_2_AR. Cells were treated with 10 ng/mL of EGF for 5 min and washed/rechallenged according to the desensitization protocol. The cell lysates were immunoprecipitated with FLAG beads. Co-IP/lysate and IP were immunoblotted with antibodies against GFP and FLAG. *p < 0.05 compared with other groups of the cells expressing WT-β_2_AR or GRK2-KO-β_2_AR (*n* = 3). **D** HEK-293 cells expressing β_2_AR were treated with either vehicle or 10 ng/mL of EGF for 20 min, followed by an increasing concentration of isoproterenol. ***p < 0.001 when compared between Veh- and EGF-treated groups at 10^−8.5^–10^−5^ M of isoproterenol (*n* = 3)

The desensitization mechanisms of GPCRs have been extensively studied using the β_2_AR receptor [2, 4]. Therefore, we tested the involvement of EGFR transactivation in the desensitization of β_2_AR. As depicted in Fig. 8A, repeated exposure to isoproterenol resulted in the desensitization of β_2_AR (comparing the Veh/Veh group with the ISO/Veh group). Pretreatment with AG1478 prevented the desensitization of β_2_AR (Fig. 8A) and inhibited the ERK activation (Fig. 8B), akin to observations made with D2-like receptors. Similar to the scenario with D_3_R, EGF treatment promoted an enhanced interaction between EGFR and β_2_AR (Fig. 8C), leading to the suppression of β_2_AR signaling (Fig. 8D). These collective findings suggest an association between β_2_AR desensitization and EGFR transactivation.

### Involvement of GRK2 and GRK6 in the desensitization of β_2_AR and the transactivation of EGFR

**Fig. 9:**
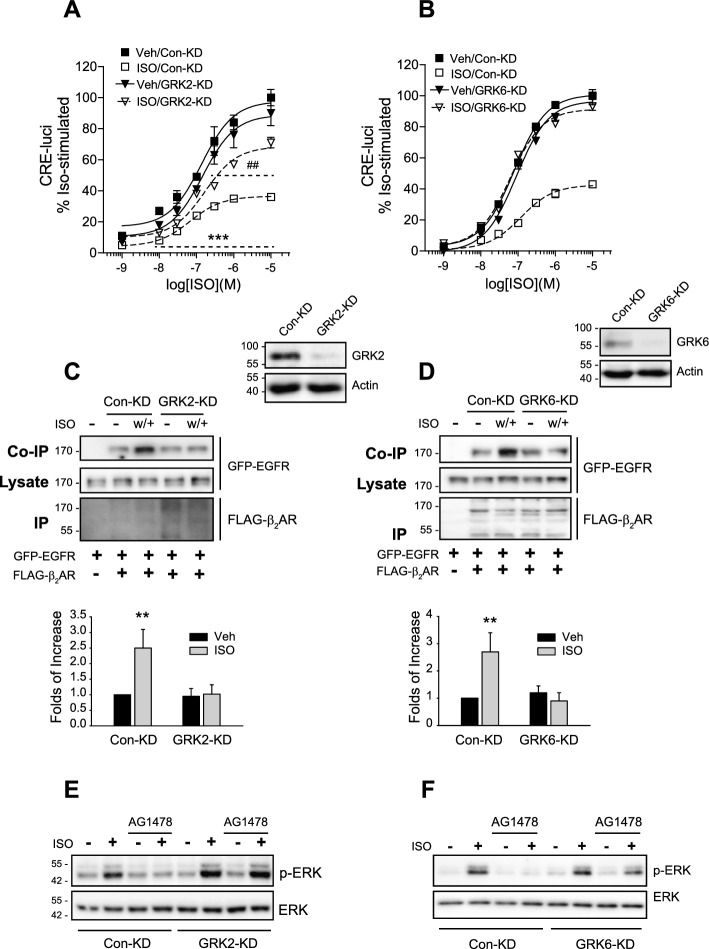
Roles of GRK2 and GRK6 in the desensitization of β_2_ adrenoceptors and its transactivation of EGFRs. **A**–**D** Cells were treated with 10 μM of isoproterenol for 5 min and washed/rechallenged to induce desensitization. **A** Con-KD and GRK2-KD HEK-293 cells were transfected with β_2_AR. ^##^*p* < 0.01 when the ISO/GRK2-KD group was compared with its vehicle-treated group; ***p < 0.001 when the ISO/Con-KD group was compared with its vehicle-treated group (*n* = 3). **B** Con-KD and GRK6-KD HEK-293 cells were transfected with β_2_AR. ***p < 0.001 when the ISO/Con-KD group was compared with other experimental groups (*n* = 3). **C** Con- and GRK2-KD HEK-293 cells were transfected with EGFR-GFP and FLAG-β_2_AR. **p < 0.01 compared with other groups of the Con-KD and GRK2-KD cells (*n* = 3). Cell lysates from Con-KD and GRK2-KD cells were immunoblotted with antibodies against GRK2 and β-actin. The knockdown efficiency of arrestin2 and arrestin3 was about 85%. **D** Con-KD and GRK6-KD HEK-293 cells were transfected with EGFR-GFP and FLAG-β_2_AR. ^##^*p* < 0.01 compared with other groups of the Con-KD and GRK2-KD cells (*n* = 3). Cell lysates from Con-KD and GRK6-KD cells were immunoblotted with antibodies against GRK6 and β-actin. The knockdown efficiency of GRK6 was about 95%. **E** Con-KD and GRK2-KD HEK-293 cells were transfected with β_2_AR. Cells were pretreated with either vehicle or 200 nM of AG1478 for 30 min, followed by treatment with 10 μM of isoproterenol for 2 min to induce ERK activation. The ratios of ISO(+)/ISO(−) were 2.3 ± 0.45 (Veh/Con-KD), 0.95 ± 0.32 (AG1478/Con-KD), 3.5 ± 0.75 (Veh/GRK2-KD), and 3.3 ± 0.60 (AG1478/GRK2-KD). AG1478/Con-KD group was significantly different from the Veh/Con-KD group (*p* < 0.05, *n* = 3) and the groups of GRK2-KD cells (*p* < 0.01, *n* = 3). **F** Con-KD and GRK6-KD HEK-293 cells were transfected with β_2_AR. Cells were treated as in **E**. The ratios of ISO(+)/ISO(−) were 2.2 ± 0.34 (Veh/Con-KD), 0.97 ± 0.26 (AG1478/Con-KD), 2.5 ± 0.45 (Veh/GRK2-KD), and 2.05 ± 0.37 (AG1478/GRK2-KD). AG1478/Con-KD group was significantly different from other groups (*p* < 0.05, *n* = 3)

A prior investigation has established the involvement of GRK6 and, to a lesser extent, GRK2 in β_2_AR desensitization [26]. Consistent with these findings, the knockdown of GRK6 and, to a lesser extent, GRK2 attenuated isoproterenol-induced β_2_AR desensitization (Fig. 9A and B) and prevented the interaction between β_2_AR and EGFR induced during desensitization (Fig. 9C and D). Additionally, the knockdown of GRK2 or GRK6 abolished the contribution of EGFR transactivation in β_2_AR-mediated ERK activation (Fig. 9E and F).

**Fig. 10:**
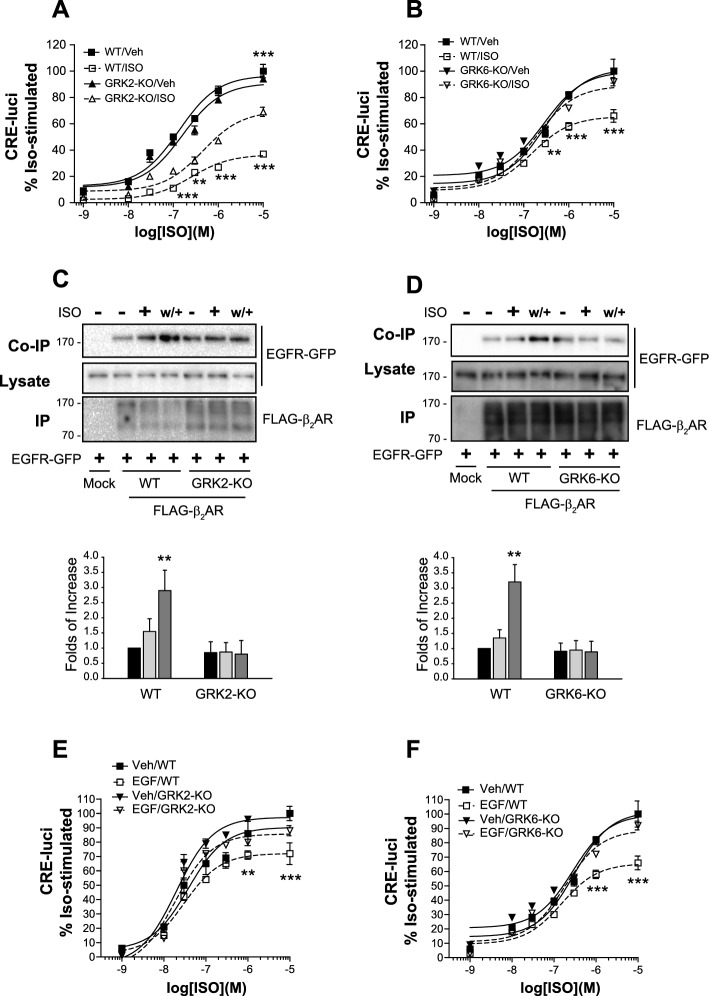
Roles of GRK2- and GRK6-mediated receptor phosphorylation in the desensitization of β_2_AR and its transactivation of EGFRs. **A**–**D** Cells were treated with 10 μM of isoproterenol for 5 min and washed/rechallenged to induce desensitization. **A** HEK-293 cells were transfected with WT-β_2_AR or GRK2-KO-β_2_AR. ISO/GRK2-KO group was significantly different from other groups (*p* < 0.001, *n* = 3) except at 3 × 10^−7^ M isoproterenol. **p < 0.01 compared with ISO/Con-KD group (*n* = 3). **B** HEK-293 cells were transfected with WT-β_2_AR or GRK6-KO-β_2_AR. **p < 0.01, ***p < 0.001 compared with other experimental groups (*n* = 3). **C** HEK-293 cells were transfected with EGFR-GFP together with WT-β_2_AR or GRK2-KO-β_2_AR. The cell lysates were immunoprecipitated with FLAG beads. Co-IP/lysate and IP were immunoblotted with antibodies against GFP and FLAG. ^#^*p* < 0.05 compared with the “+/WT” group; **p < 0.01 compared with other groups of the cells expressing WT-β_2_AR or GRK2-KO-β_2_AR except the “ + /WT” group (*n* = 3). **D** HEK-293 cells were transfected with EGFR-GFP together with WT-β_2_AR or GRK6-KO-β_2_AR. ^##^*p* < 0.01 compared with other groups of the cells expressing WT-β_2_AR or GRK6-KO-β_2_AR (*n* = 3). **E** HEK-293 cells were transfected with WT- or GRK2-KO-β_2_AR. Cells were pretreated with either vehicle or 10 ng/mL of EGF for 20 min. **p < 0.01, ***p < 0.001 when the EGF/WT group was compared with other groups of the cells expressing WT-β_2_AR and GRK2-KO-β_2_AR; ^##^*p* < 0.01, ^###^*p* < 0.001 when EGF/WT group was compared to Veh/GRK2-KO or EGF/GRK2-KO group (*n* = 3). **F** HEK-293 cells were transfected with WT- or GRK6-KO-β_2_AR. Cells were pretreated with either vehicle or 10 ng/mL of EGF for 20 min. ***p < 0.001 when the EGF/WT group was compared with other groups of the cells expressing WT-β_2_AR or GRK6-KO-β_2_AR (*n* = 3). (**E**) or GRK6-KO-β_2_AR (**F**). Cells were treated with 10 ng/mL of EGF for 20 min. **p < 0.01, ***p < 0.001 compared with Veh/WT group (*n* = 3)

To validate the roles of GRK2- and GRK6-mediated receptor phosphorylation in β_2_AR desensitization and the interaction between β_2_AR and EGFR, we mutated the consensus phosphorylation sites of GRK2 (T360, S364, S396, S401, S407, S411) or GRK6 (S355, S356) in β_2_AR, as previously outlined [26]. These mutants were denoted as GRK2-KO-β_2_AR and GRK6-β_2_AR, respectively. Both mutants exhibited significantly reduced desensitization compared with WT-β_2_AR (Fig. 10A and B). Importantly, neither mutant interacted with EGFR under desensitization conditions (Fig. 10C and D).

Finally, direct stimulation of EGFR inhibited cAMP production through β_2_AR. However, the inhibition of cAMP production induced by EGF was noticeably weaker in GRK2-KO-β_2_AR or GRK6-KO-β_2_AR compared with WT-β_2_AR (Fig. 10E and F).

These results imply that GRK6 and GRK2 contribute to β_2_AR desensitization by facilitating the transactivation of EGFR.

## Discussion

The widely accepted steric hindrance (or two-step) model of GPCR desensitization suggests that G proteins and arrestins competitively bind to the same regions of agonist-bound receptors, resulting in the uncoupling of receptors from G proteins. However, subsequent research on D2-like receptors has revealed that the desensitization of GPCRs involves more complex cellular processes than originally proposed. For instance, studies have shown that the deubiquitination of arrestins plays a crucial role in the desensitization of certain receptors, such as D2-like receptors and β_2_AR [10, 11, 35]. The regulatory cascade involving Src, PDK1, Akt, and USP33 is implicated in the process of arrestin deubiquitination.

The main objective of this study was to identify the cellular components that link the activation of GPCRs and the recently proposed desensitization cascade [11]. To meet the criteria of an intermediate linker, the associated factor must be activated by agonistic stimulation of receptors that can undergo desensitization. Furthermore, this intermediate linker should be capable of directly stimulating the cellular components responsible for GPCR desensitization mentioned earlier.

**Fig. 11:**
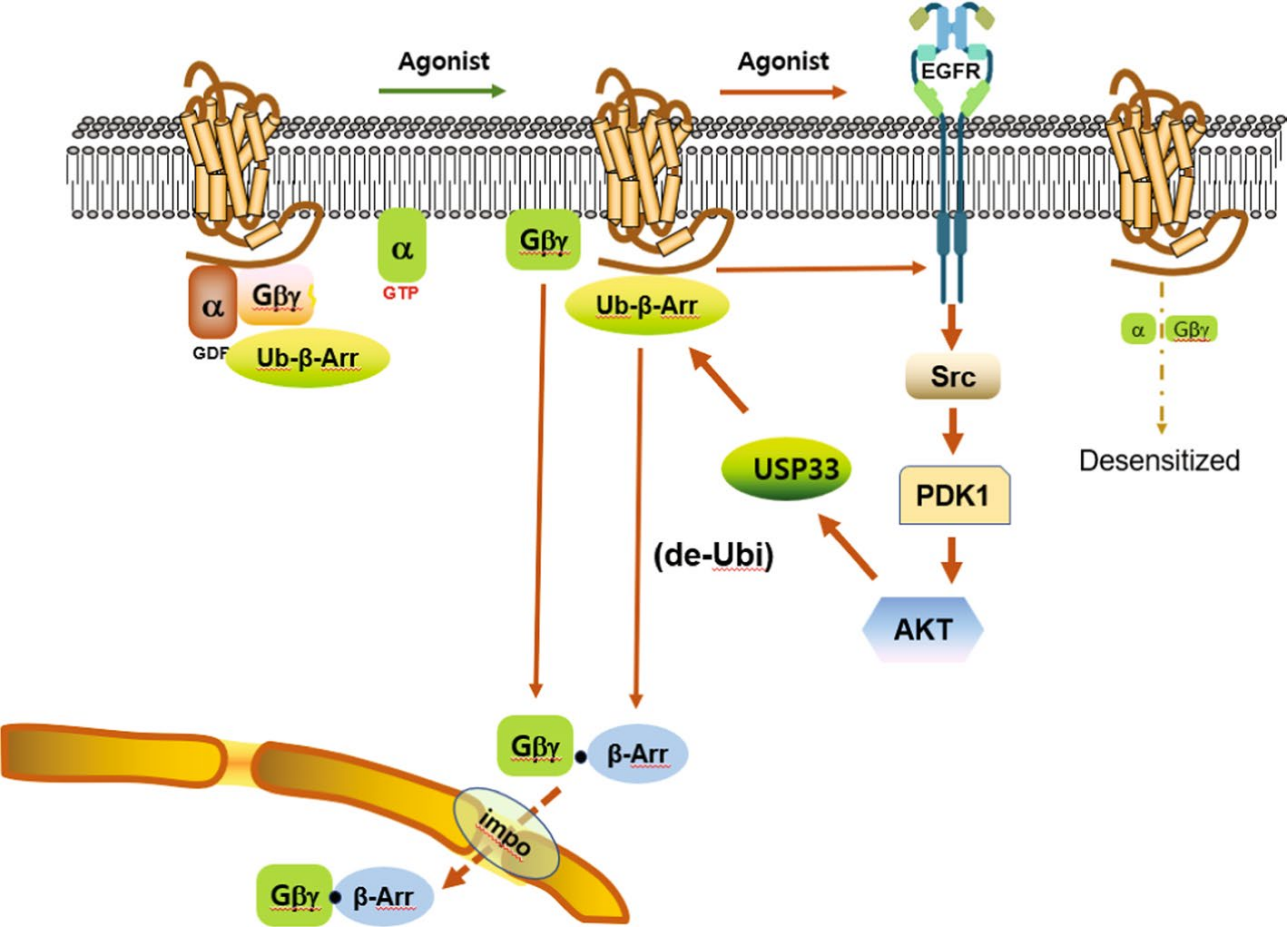
Diagram illustrating the molecular processes involved in GPCR desensitization via transactivation of EGFR. The content presented in this diagram aligns with the newly proposed concept of the GPCR desensitization hypothesis, as described in recent literature [11]. In the resting state, Mdm2 relocates to the cytoplasm, where it continually adds ubiquitin molecules to arrestins. When desensitization occurs, the Gα and Gβγ subunits detach from the receptor. Concurrently, the receptor triggers signaling through Src, activating PDK1 and subsequently Akt via phosphorylation at S241 and T308, respectively. Active Akt then interacts with USP33 to enhance its enzyme activity, leading to the removal of ubiquitin molecules from arrestins. Once deubiquitinated, arrestins bind with Gβγ and migrate to the nucleus through the importin complex, effectively sequestering Gβγ from both the receptor and Gα. This action diminishes the efficiency of receptor signaling, resulting in desensitization. In this study, we add that a cascade of regulatory events involved in this innovative GPCR desensitization process is initiated through transactivation between GPCR and EGFR

The conclusions of this study on these questions are shown in Fig. 11. The overall cascade for GPCR desensitization is cited from a recent publication [11], and what was added through this study is that the connection between the receptor and the downstream cascade that occurs through transactivation of EGFR.

While it cannot be definitively ruled out that other cellular components may also be involved, it is evident that Src plays a central role in connecting EGFR to downstream signaling pathways. This inference is substantiated by numerous research findings. For instance, c-Src interacts with EGFR and colocalizes with it within lipid rafts, facilitating c-Src signaling, including c-Src-dependent activation of PI3K/Akt [43–45]. In turn, c-Src phosphorylates EGFR, thereby triggering downstream signaling to modulate EGF-induced mitogenesis [46–50].

The EGF-related findings in this study were obtained independently of EGFR coexpression. However, prior research has shown that coexpression of EGFR leads to tyrosine phosphorylation of GRK2, thereby activating GRK2 [51]. This activated GRK2 interacts with D_3_R, resulting in the inhibition of signaling and subsequent downregulation. Similar observations have been documented for the δ-opioid receptor [52]. Notably, in the homologous desensitization of D_3_R, GRK2 does not have a role [10], indicating a distinct mechanism from the one elucidated in this study. Considering these factors, the regulatory mechanism involving GRK2 tyrosine phosphorylation differs qualitatively from desensitization induced by arrestin deubiquitination [11] and is likely associated with a stronger activation of EGFR. This implies that varying regulatory mechanisms may be at work depending on the level of EGFR expression in a given tissue.

GPCR-mediated EGFR transactivation has been recognized as a mechanism that enhances signaling diversity. Ligand-stimulated GPCRs result in matrix metalloproteases (MMPs)-mediated proteolytic cleavage of EGFR ligand, which then binds to the EGFR. Various relay or scaffolding molecules, such as arrestins, Gβγ, reactive oxygen species (ROS), PKCα, PLCβ, and Ca^2+^ ions act as regulators of MMPs. In our study, GRK2 and GRK6 were found to be involved in the transactivation of EGFR by β_2_AR, whose roles in the interaction between GPCR and EGFR have not been previously reported.

Therefore, it is worth testing the involvement of EGFR transactivation in diverse desensitization mechanisms that cannot be adequately explained by existing known mechanisms. For example, Src and/or Gβγ in the desensitization of the δ-opioid receptor [53–55], ROS with the μ-opioid receptor [56], and GRK2 with Gq-coupled receptors [57].

GPCR-mediated EGFR transactivation is assumed to provide a coordinating input that enables different GPCRs to effectively modulate physiological and pathological processes [58]. For example, βAR-mediated EGFR transactivation has been suggested to serve as a cardioprotective pathway [59, 60], which now can be attributed to the desensitization of βARs via transactivation of EGFR. A similar case was reported in the angiotensin type 1 receptor, where pretreatment with EGF has been shown to inhibit the signaling of the angiotensin type 1 receptor [61]. Consequently, understanding the molecular mechanisms involved in GPCR-mediated transactivation of EGFR in relation to GPCR desensitization could offer more insightful explanations for previously unexplained EGFR-related physiological and pathological outcomes.

## Conclusions

In this study, we explored how repeated agonistic stimulation of GPCRs initiates the desensitization cascade from perspectives beyond the conventional steric hindrance model. On the basis of preliminary experiments on the signaling cascade involved in GPCR desensitization, we hypothesized that EGFR mediates GPCR desensitization. Our results indicated that the homologous desensitization of D2-like receptors and β_2_AR can be explained by GPCR-mediated transactivation of EGFR. Given the importance of receptor desensitization in determining drug efficacy, the new regulatory principles uncovered in this study are valuable for both therapeutic applications and the development of new drugs.

## Supplementary Information

Supplementary Material 1

## Data Availability

The materials are available from the corresponding author on request.

## Abbreviations

ADMA: A disintegrin and metalloprotease
Akt: Protein kinase B
β_2_AR: β_2_ Adrenergic receptor
D_2_R: Dopamine D_2_ receptor
DA: Dopamine
EGFR: Epidermal growth factor receptor
ERK: Extracellular signal-regulated kinase
GPCR: G protein-coupled receptor
GRK: G protein-coupled receptor kinase
ISO: Isoproterenol
MMP: Matrix metalloprotease
PDK1: 3-Phosphoinositide-dependent protein kinase 1
QUIN: Quinpirole
RTK: Receptor tyrosine kinase
Ub: Ubiquitin
USP33: Ubiquitin-specific peptidase 33
Src: Proto-oncogene tyrosine-protein kinase
