# Supplementary material for: Transactivation of the EGF receptor as a novel desensitization mechanism for G protein-coupled receptors, illustrated by dopamine D2-like and β2 adrenergic receptors
Source: Cell Mol Biol Lett. 2024 Oct 28;29:132. doi: 10.1186/s11658-024-00652-z (PMC11514929; doi:10.1186/s11658-024-00652-z)
Supplement: Supplementary file 1 — Supplementary Material 1 [file 11658_2024_652_MOESM1_ESM.pdf]

Transactivation of the EGF receptor as a novel desensitization mechanism for G protein-coupled receptors, illustrated by dopamine D2-like and β_2 adrenergic receptors

Dooti Kundu, Xiao Min, Shujie Wang, Lulu Peng, Xinru Tian, Mengling Wang, Kyeong-Man Kim*

Figure S1. Characterizing the selectivity of AG1478 for EGFR.

HEK-293 cells were transfected with FLAG-Akt and EGFR (A-D) or PDGFR (E, F). The next day, cells were starved in the serum-free media for 18 h with serum-free media. Cells were pretreated with AG1478 in a time-dependent (A, C) or dose-dependent manner (B, D, E, F). Cells were treated with 10 ng/mL EGF for 5 min and washed/re-challenged with EGF for 5 min to induce desensitization (w/+). Cell lysates were immunoprecipitated with FLAG beads and IPs were immunoblotted with antibodies against p-Akt (T308, 1:1,000 dilutions)/FLAG (1:1,000 dilutions)(A, B, E) or p-ERK2/ERK2 (1:1,000 dilutions)(C, D, F).
(**A**, **C**) Cells were pretreated with vehicle or 200 nM AG1478 between 0 to 30 min.
(**B**, **D**, **E**, **F**) Cells were pretreated with vehicle or AG1478 (10, 100, 200 nM) for 30 min.

Fig. S1

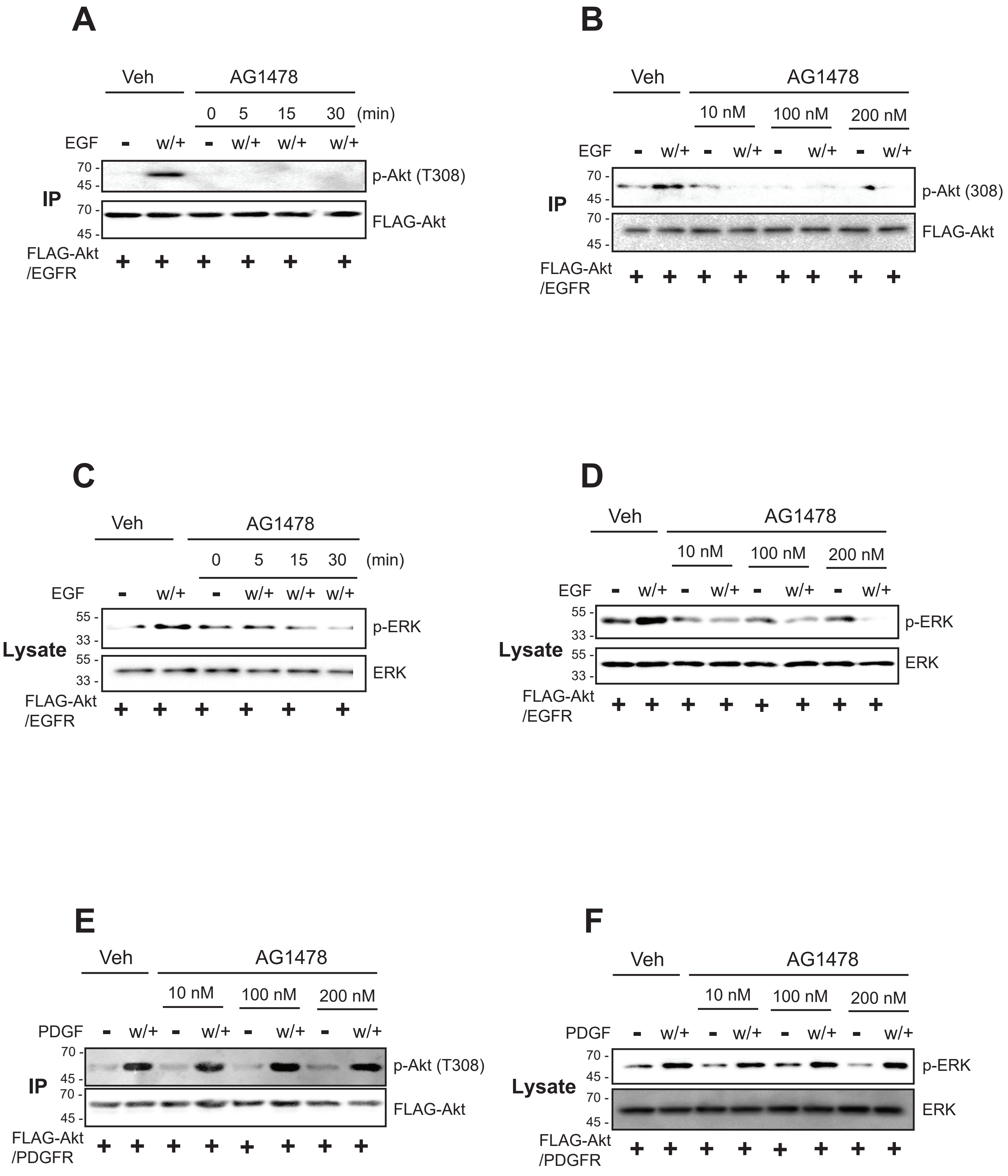
A
Veh
AG1478
0 5 15 30 (min)
EGF - w/+ - w/+ w/+ w/+
70 -
45 -
p-Akt (T308)
IP
70 -
45 -
FLAG-Akt
FLAG-Akt /EGFR + + + + + +
B
Veh
AG1478
10 nM
100 nM
200 nM
EGF - w/+ - w/+ - w/+ - w/+
70 -
45 -
p-Akt (308)
IP
70 -
45 -
FLAG-Akt
FLAG-Akt /EGFR + + + + + + + +
C
Veh
AG1478
0 5 15 30 (min)
EGF - w/+ - w/+ w/+ w/+
55 -
33 -
p-ERK
Lysate
55 -
33 -
ERK
FLAG-Akt /EGFR + + + + + +
D
Veh
AG1478
10 nM
100 nM
200 nM
EGF - w/+ - w/+ - w/+ - w/+
55 -
33 -
p-ERK
Lysate
55 -
33 -
ERK
FLAG-Akt /EGFR + + + + + + + +
E
Veh
AG1478
10 nM
100 nM
200 nM
PDGF - w/+ - w/+ - w/+ - w/+
70 -
45 -
p-Akt (T308)
IP
70 -
45 -
FLAG-Akt
FLAG-Akt /PDGFR + + + + + + + +
F
Veh
AG1478
10 nM
100 nM
200 nM
PDGF - w/+ - w/+ - w/+ - w/+
70 -
45 -
p-ERK
Lysate
70 -
45 -
ERK
FLAG-Akt /PDGFR + + + + + + + +
